# Guidelines on Placenta Accreta Spectrum Disorders: A Systematic Review

**DOI:** 10.1001/jamanetworkopen.2025.21909

**Published:** 2025-07-18

**Authors:** Giulia Bonanni, Maria C. Lopez-Giron, Lisa Allen, Karin Fox, Robert M. Silver, Sebastian R. Hobson, Albaro J. Nieto-Calvache, Sally Collins, Miroslaw Wielgos, Eric Jauniaux, Helena C. Bartels, Loïc Sentilhes, John Kingdom, Frederic Chantraine, Joseph R. Wax, Alison Cahill, Alfred Abuhamad, Diogo Ayres de-Campos, Kjersti Aagaard, Amir A. Shamshirsaz, Scott A. Shainker, Alireza A. Shamshirsaz

**Affiliations:** 1 Fetal Care and Surgery Center, Division of Fetal Medicine and Surgery, Boston Children’s Hospital, Harvard Medical School, Boston, Massachusetts; 2 Department of Women, Children, and Public Health Sciences, IRCCS Agostino Gemelli University Polyclinic Foundation, Catholic University of the Sacred Heart, Rome, Italy; 3 Facultad de Ciencias de la Salud, Universidad Icesi, Cali, Colombia; 4 Section of Pediatric and Adolescent Gynecology, The Hospital for Sick Children, Department of Obstetrics and Gynecology, Temerty Faculty of Medicine, University of Toronto, Toronto, Ontario, Canada; 5 Department of Obstetrics and Gynecology, Mount Sinai Hospital, Toronto, Ontario, Canada; 6 Department of Obstetrics and Gynecology, University of Texas Medical Branch, Galveston; 7 Department of Obstetrics and Gynecology, University of Utah Health, Salt Lake City; 8 Division of Maternal-Fetal Medicine, Department of Obstetrics and Gynaecology, Mount Sinai Hospital, University of Toronto, Toronto, Ontario, Canada; 9 Clinica de Espectro de Acretismo Placentario, Fundacion Valle de Lili, Cali, Colombia; 10 Nuffield Department of Women’s and Reproductive Health, University of Oxford, Oxford, UK; 11 Fetal Medicine Unit, John Radcliffe Hospital, Oxford, UK; 12 Department of Obstetrics and Perinatology, National Medical Institute of the Ministry of Interior and Administration, Warsaw, Poland; 13 EGA Institute for Women’s Health, Faculty of Population Health Sciences, University College London, London, UK; 14 Department of Obstetrics and Gynaecology, National Maternity Hospital, University College Dublin School of Medicine, Dublin, Ireland; 15 Department of Obstetrics and Gynecology, Bordeaux University Hospital, Bordeaux, France; 16 Department of Obstetrics and Gynecology, Mount Sinai Hospital, University of Toronto, Toronto, Ontario, Canada; 17 Service of Obstetrics and Gynecology, Citadelle Hospital, CHU Liege, Liege, Belgium; 18 Division of Maternal-Fetal Medicine, Maine Medical Center, Portland; 19 Department of Women’s Health, University of Texas at Austin, Dell Medical School, Austin; 20 Division of Maternal-Fetal Medicine, Department of Obstetrics and Gynecology, Eastern Virginia Medical School, Norfolk; 21 Lisbon Medical School, University of Lisbon, Santa Maria University Hospital, Lisbon, Portugal; 22 HCA Healthcare Research Institute, HCA Healthcare, Nashville, Tennessee; 23 Texas Maternal Fetal Medicine, HCA Healthcare, Houston; 24 Division of Maternal-Fetal Medicine, Department of Obstetrics and Gynecology, Baylor College of Medicine, Houston, Texas; 25 Department of Obstetrics and Gynecology, Beth Israel Deaconess Medical Center, Harvard Medical School, Boston, Massachusetts; 26 Department of Obstetrics, Gynecology, and Reproductive Biology, Harvard Medical School, Boston, Massachusetts

## Abstract

**Importance:**

Placenta accreta spectrum (PAS) is a complex, life-threatening condition that demands a multidisciplinary approach involving obstetrics, maternal-fetal medicine, and various surgical and medical specialties. Effective management relies on multispecialty collaboration and consensus, supported by standardized protocols, to optimize outcomes, guide informed clinical decisions, and mitigate the risks associated with PAS.

**Objective:**

To examine clinical practice guidelines for PAS inclusive of high-income countries and low- to middle-income countries (LMICs) identifying areas of consensus and gaps in guidance.

**Evidence Review:**

A comprehensive search of PubMed, GIN Library, and ECRI Guidelines Trust identified all PAS-related clinical practice guidelines published from January 1, 2014, to January 31, 2024. Additional searches included professional societies’ designated websites and cited references. Two independent reviewers screened the guidelines, resolving conflicts through cross-referencing. Initially, 2 independent reviewers provided structured review and feedback to refine, correct, or highlight areas of consensus, disagreement, or insufficient evidence. Any instances of nonagreement were adjudicated by majority panel agreement, arising from a panel of 15 to 18 experts, all authors of PAS guidelines. Agreement scores for each recommendation area (eg, epidemiology, diagnosis, and antenatal management) were categorized as high agreement (≥75%), poor consensus (<50% or ≥30% insufficient evidence), and high levels of insufficient evidence (≥50% of recommendations with insufficient evidence) based on a priori score criteria.

**Findings:**

A total of 14 guidelines from 18 articles from national and international societies were included. High agreement was noted in areas such as specialized expertise (100%), antenatal management (88.9%), diagnosis (76.9%), and epidemiology (75.0%). Poor consensus characterized cesarean hysterectomy management (38.5% insufficient evidence and 23.0% disagreement), conservative techniques (33.3% insufficient evidence and 11.1% disagreement), and fertility counseling (30.0% insufficient evidence and 10.0% disagreement). Despite the high risk of anemia, consensus was lacking on iron supplementation strategies. Recommendations for thromboembolism prevention varied, with some guidelines favoring pharmacologic interventions and others advocating for nonpharmacologic measures. Hemorrhage management and postnatal management recommendations, including iron supplementation and thromboembolism prevention, were characterized by high levels of insufficient evidence (55.6% and 57.1%, respectively). Only 1 article (5.6%) specifically addressed LMICs, highlighting substantial underrepresentation.

**Conclusions and Relevance:**

This systematic review of PAS guidelines identified significant discrepancies and insufficient evidence in key aspects of care. The findings underscore the urgent need for further research and quality measures to enhance standardized approaches and improve patient outcomes. The limited availability of recommendations applicable to LMICs highlights the critical need for tailored guidance that accounts for resource constraints and clinical access challenges unique to these settings.

## Key Points

**Question:**

What are the levels of agreement and gaps in guidelines for placenta accreta spectrum disorders across different income settings?

**Findings:**

This systematic review of 14 guidelines from national and international societies found high agreement on specialized expertise needed and antenatal management but poor consensus on management, including cesarean hysterectomy and conservative surgical techniques. Many topics with insufficient evidence were identified, especially for postnatal care, but only 1 guideline addressed specific needs for low- and middle-income countries.

**Meaning:**

There are substantial discrepancies and evidence gaps in placenta accreta spectrum guidelines worldwide, suggesting that increased research and guideline standardization are needed to improve PAS management globally.

## Teaser

This systematic review identifies areas of consensus and highlights gaps in guidance on management of placenta accreta spectrum disorders across high- and low- to middle-income countries.

## Introduction

Placenta accreta spectrum (PAS) is a complex, life-threatening condition that demands a comprehensive multidisciplinary approach. This approach involves not only obstetrics and maternal-fetal medicine but also collaboration with various surgical specialties, including urology, surgical oncology, and advanced gynecologic surgery, and medical specialties, including anesthesiology, critical care, transfusion medicine, and hematology.^1^ Coordinated efforts are crucial to address the significant morbidity and mortality associated with placental tissue invading adjacent pelvic organs, often causing severe hemorrhage.^2,3^ Effective management requires adherence to standardized clinical practice guidelines (CPGs) to inform clinical decisions and optimize outcomes.

Although previous reviews^4,5,6^ have provided some comparisons between the main guidelines on PAS, a gap remains in systematically evaluating discrepancies and evidence across all CPGs, particularly concerning variations between high-income countries and low- to middle-income countries (LMICs). Our systematic review aims to address this gap by providing a comprehensive, methodologically rigorous comparative analysis of all published national and international PAS guidelines. Using an extensive search strategy, systematic data extraction, assessment by a panel of global PAS experts, and a specific focus on recommendations for LMICs, we aimed to identify areas of significant intersociety variation and highlight gaps in evidence or guidance to inform future research and improve clinical practice.

## Methods

### Eligibility Criteria and Search Strategy

This review adheres to the relevant sections of the Preferred Reporting Items for Systematic Reviews and Meta-analyses (PRISMA) reporting guideline, supplemented by the Synthesis Without Meta-analysis (SWiM) extension.^7^ This study was exempt from institutional review board review under exemption category 45 CFR §46.104(d).

We included all CPGs published between January 1, 2014, and January 31, 2024, that focused on PAS or provided pertinent recommendations within the context of cesarean delivery (CD) or late preterm bleeding. The decision to focus on this 10-year range was made to ensure the inclusion of the most current and relevant guidelines, reflecting up-to-date clinical practices and advancements in the management of PAS.

Guidelines were sourced through a comprehensive approach, including (1) examination of relevant CPGs accessible on professional societies’ websites; (2) systematic search of PubMed, GIN Library, and ECRI Guidelines Trust, using predefined keywords (Medical Subject Heading [MeSH] and title and abstract [TIAB], including placenta accreta (MeSH) OR *placenta accreta* (TIAB) OR *placenta increta* (TIAB) OR *placenta accreta spectrum* (TIAB) OR *PAS* (TIAB) OR *morbidly adherent placenta* (TIAB) AND *practice guideline* (MeSH) OR *clinical guidelines* (TIAB) OR *clinical practice guidelines* (TIAB); and (3) examination of cited references from relevant CPGs. Two independent reviewers (G.B. and M.C.L.-G.) screened all guidelines. Geographic or language restrictions were not imposed. For multiple versions of a guideline, only the most recent was included to ensure relevance and accuracy.

### Data Extraction

Two independent reviewers (G.B. and M.C.L-G.) screened included CPGs to extract relevant recommendations, resolving conflicts by cross-checking the original articles. A panel of 15 to 18 experts, all authors of PAS guidelines, reviewed the extracted recommendations via 2 SurveyMonkey rounds (SurveyMonkey Inc), providing open-ended feedback to (1) comment on, correct, or revise recommendations they had previously authored and (2) highlight areas of disagreement or insufficiency. eTables 1 to 3 in Supplement 1 summarize the characteristics of participating experts.

### Statistical Analysis

After data extraction, areas of consensus or disagreement among guidelines were identified through subjective analysis by 2 experts (Scott A. Shainker and Alireza A. Shamshirsaz). Each topic of recommendation was classified as representing agreement, disagreement, or insufficient evidence. Conflicts were reviewed and resolved by the expert panel, with final decisions determined by majority consensus. eTables 4 to 8 in Supplement 1 present the full set of recommendations for each topic, along with the corresponding agreement assessment.

To facilitate comparison across the PAS guidelines, we conducted a comparative analysis by visualizing agreement levels across different recommendations. For each recommendation area (eg, epidemiology, diagnosis, and management), a percentage score reflecting guidelines agreement was calculated using R programming language, version 4.4.1 (R Foundation for Statistical Computing). The agreement levels were categorized as high agreement (≥75% of recommendations aligned), poor consensus (<50% agreement or ≥30% of recommendations with insufficient evidence), or high levels of insufficient evidence (≥50% of recommendations with insufficient evidence). Because no robust automated method for comparing CPGs is currently available in the literature,^8^ this approach to assigning percentage scores relied on input from experts. Agreement was achieved through expert consensus, ensuring the method was tailored to the unique characteristics of our data. To visually represent the agreement levels, a heatmap was generated using the ComplexHeatmap package, version 2.22.0 in R software, version 4.4.1 (R Project for Statistical Computing).^9^ This heatmap provides a structured overview of guideline consistency, highlighting areas of high agreement, poor consensus, and insufficient evidence, thereby identifying gaps in the guidelines.

## Results

### Description of the CPGs

**Figure 1.**
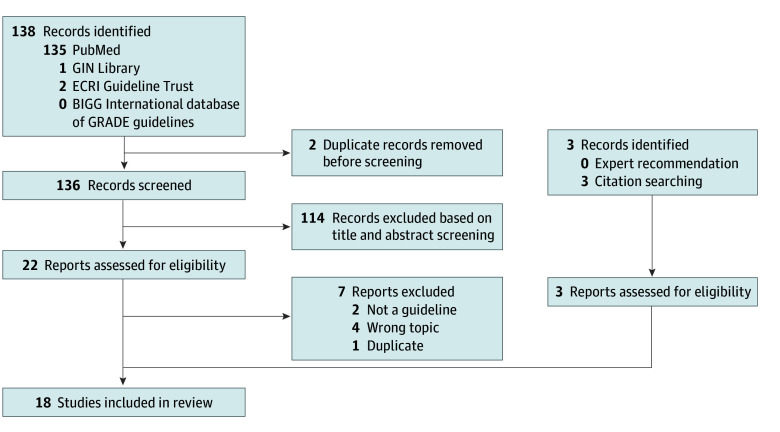
PRISMA Flow Diagram of the Study Selection Process

Table 1 presents the main characteristics of the included CPGs. Our review yielded 18 articles published within the past decade from 14 national or international societies (Figure 1). Among these, 14 (77.8%) primarily addressed PAS,^10,11,12,13,14,15,16,17,18,19,20,21,22,23^ whereas 4 (28.6%) predominantly concentrated on managing CD or postpartum hemorrhage with pertinent recommendations.^24,25,26,27^ Nine of these 18 manuscripts (50.0%) were published since 2019. Only 1 article (5.6%) included recommendations specific to LMICs throughout all 5 of its articles.^10,16,17,18,22^ Table 2 summarizes areas of consensus, disagreement, and insufficient evidence.

**Table 1.** Summary and Categorization of Included Clinical Guidelines

Study	Society	Country	Title	Year (last update)	Scope
Cahill et al^12^	ACOG and SMFM	US	Placenta accreta spectrum	2018 (reaffirmed 2021)	National
Shainker et al^23^	SMFM, AIUM, ACR, and GOHO^a^	US	Special Report of the SMFM Placenta Accreta Spectrum Ultrasound Marker Task Force	2021	National
Annecke et al^24^	AWMF	Germany	Peripartale Blutungen, Diagnostik und Therapia	2022	National
Sentilhes et al^25^	CNGOF	France	Postpartum hemorrhage: guidelines for clinical practice from the French College of Gynaecologists and Obstetricians in collaboration with the French Society of Anesthesiology and Intensive Care	2016	National
Nieto-Calvache et al^19^	FECOLSOG	Colombia	Colombian Consensus on the Treatment of Placenta Accreta Spectrum	2022	National
Jauniaux et al,^16,17,18^ Allen et al,^10^ and Sentilhes et al^22^	FIGO^b^	International	FIGO consensus guidelines on placenta accreta spectrum disorders	2018	International
Collins et al^13^	IS-PAS	International	Evidence-based guidelines for the management of abnormally invasive placenta: recommendations from the International Society for Abnormally Invasive Placenta	2019	International
	MSPE^21^	Ecuador	Anomalías de inserción placentaria y vasos sanguíneos fetales	2017	National
	NICE^26^	England	Cesarean birth NICE guideline	2024	National
Bartels et al^11^	NWIHP and IOG	Ireland	National Clinical Practice Guideline: Diagnosis and Management of Placenta Accreta Spectrum	2022	National
Wielgos et al^27^	PSGO	Poland	Recommendation of the PSGO regarding cesarean deliveries	2018	National
	RANZCOG^20^	Australia and New Zealand	Placenta accreta spectrum	2023	National
Jauniaux et al^15^	RCOG	UK	Placenta previa and placenta accreta: diagnosis and management	2018	National
Hobson et al^14^	SOGC	Canada	Screening, diagnosis, and management of placenta accreta spectrum disorders	2019	National

Abbreviations: ACOG, American College of Obstetricians and Gynecologists; ACR, American College of Radiologists; AIUM, American Institute of Ultrasound in Medicine; AWMF, Association of Scientific Medical Societies in Germany; CNGOF, Collège National des Gynécologues et Obstétriciens Français; FECOLSOG, Federación Colombiana de Obstetricia y Ginecología; FIGO, Fédération Internationale de Gynécologie et d’Obstétrique; GOHO, Gottesfeld Hohler Memorial Society; IOG, Institute of Obstetricians and Gynaecologists; IS-PAS, International Society for Placenta Accreta Spectrum; ISUOG, International Society of Ultrasound In Obstetrics and Gynecology; MSPE, Ministerio de Salud Publica del Ecuador; NICE, National Institute for Health and Care Excellence; NWIHP, National Women and Infants Health Programme; PSGO, Polish Society of Gynecologists and Obstetricians; RANZCOG, Royal Australian and New Zealand College of Obstetricians and Gynaecologists; RCOG, Royal College of Obstetricians and Gynaecologists; SMFM, Society of Maternal-Fetal Medicine; SOGC, Society of Obstetricians and Gynaecologists of Canada; SRU, Society of Radiologists in Ultrasound.

^a^ ACOG and ISUOG support this document. SRU approves this document.

^b^ FIGO guidelines are presented across 5 articles, covering the following topics: introduction, epidemiology, prenatal diagnosis and screening, nonconservative surgical management, and conservative management.

**Table 2.** Summary of Recommendations: Areas of Strong Consensus, Disagreement, and Insufficient Evidence

Section	Areas of consensus	Areas of disagreement	Areas of insufficient evidence
Epidemiology	Previous CD is a significant risk factor for PAS Risk increases with the number of CDs PAS risk is associated with previous myomectomy and minor uterine procedures, placenta previa, and low-lying placenta	None	Reporting of other risk factors, such as maternal age, multiparity, multiple pregnancies, IVF, and smoking, varies across guidelines Inconsistent recommendations on PAS risk counseling during preconception
Prenatal screening and diagnosis	Antenatal screening helps in optimizing management and outcomes The screening should be done on patients with risk factors, especially prior CD and placenta previa Ultrasonography is the recommended first-line screening modality, TVUS enhances visualization, and color flow Doppler ultrasonography is recommended MRI can be valuable in specific cases	MRI: contrast media use Diagnostic biomarkers use	MRI: timing Lack of consensus on screening patients with anterior low-lying placenta
Antenatal management	Elective CD is recommended; timing of delivery is consistently reported as 34-35 weeks, though some guidelines recommend up to 37 weeks Corticosteroids recommended if risk of delivery before 35 weeks Hospital admission for symptomatic patients	None	Lack of clear guidance on optimal surveillance timeframe Insufficient evidence to support routine serial growth scans Use of pharmacologic thromboprophylaxis
Expertise required for managing PAS	Care should be provided in specialist centers with specific expertise and resources; early referral to specialized centers is recommended MDT availability on site is required Specialist centers should have significant experience in PAS management, surgical capacity, critical care facilities, and continuous staff training	None	Insufficient evidence supporting the need for certain surgical specialists (colorectal and vascular surgeons) in the MDT
Cesarean hysterectomy management	Modified dorsal lithotomy position for delivery Preoperative or intraoperative ultrasonography recommended to map placental edges Uterine incision at sufficient distance from the placenta Avoid placental removal to prevent severe bleeding Use of tranexamic acid to prevent or manage hemorrhage is recommended	Choice between general and regional anesthesia. Skin incision choice Use of preoperative or intraoperative balloon occlusion catheters	Lack of evidence supporting total vs subtotal hysterectomy. Insufficient evidence on planned delayed hysterectomy Lack of evidence on deliberate cystotomy and excision of the involved bladder Routine use of bilateral internal iliac artery ligation and ureteric stent placement
Conservative management	Comprehensive counseling recommended for patients considering fertility preservation. Should include feasibility and risks Forcible manual placental removal is not recommended “Leaving the placenta in situ” is generally accepted in selected cases. “One-step conservative surgery” is possible for focal central disease Methotrexate adjuvant treatment and preventive surgical or radiological uterine devascularization are not recommended Use of tranexamic acid to prevent or manage hemorrhage is recommended Postoperative antibiotic therapy is recommended, especially if placenta left in situ	Management after intrapartum diagnosis Quantified risk of recurrent PAS is not consistent Gentle attempted placenta removal is variably accepted depending on the suspected presence of PAS Recommendations on uterotonics are not consistent	Insufficient evidence on future pregnancy rates after conservative management Insufficient guidance on mentioning risks of CD scar pregnancy, ICU admission, or death during counseling Insufficient guidance on triple-P procedure, aortic balloon vs bilateral iliac occlusion Lack of consensus on monitoring protocols for patients opting for conservative management Hemorrhage management: insufficient guidance on manual vascular compression, intrauterine tamponade, internal iliac artery ligation, and use of hemostatic agents or procoagulants
Postnatal care	Placental pathological analysis for final diagnosis is recommended Creation of comprehensive patient records is recommended Postnatal referrals are recommended, especially to social work and perinatal mental health services	None	Insufficient guidance on postnatal analgesia protocols Lack of consensus on postnatal iron supplementation strategies Guidance on thromboembolism prevention strategies varies, with some guidelines recommending pharmacologic interventions and others emphasizing nonpharmacologic measures

Abbreviations: CD, cesarean delivery; IVF, in vitro fertilization; MDT, multidisciplinary team; MRI, magnetic resonance imaging; PAS, placenta accreta spectrum; TVUS, transvaginal ultrasonography.

### Epidemiology and Prenatal Diagnosis

#### Epidemiology

**Figure 2.**
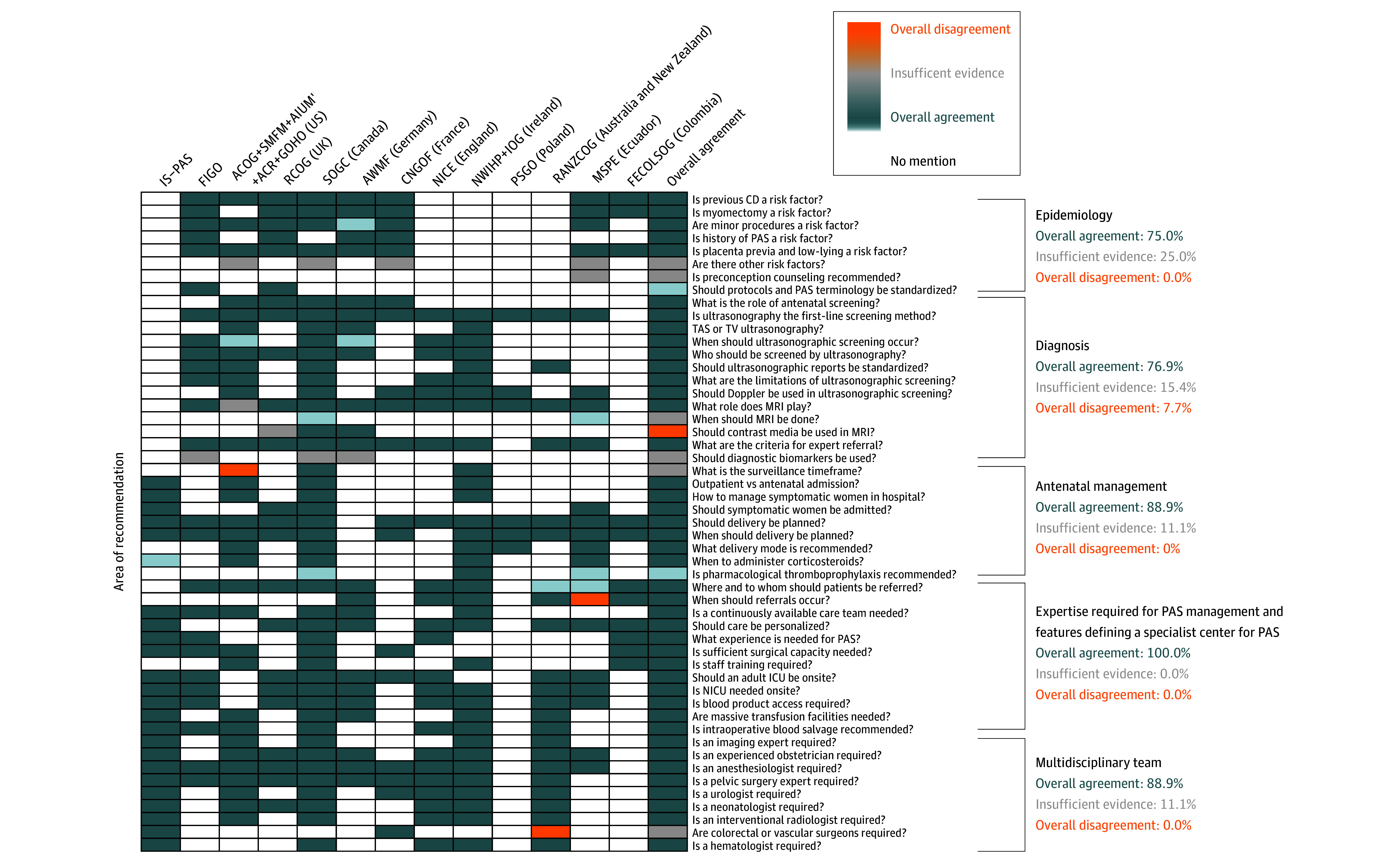
Epidemiology and Prenatal Diagnosis and Management ACOG indicates American College of Obstetricians and Gynecologists; ACR, American College of Radiology; AIUM, American Institute of Ultrasound in Medicine; AWMF, Association of Scientific Medical Societies in Germany; CD, cesarean delivery; CNGOF, Collège National des Gynécologues et Obstétriciens Français; FECOLSOG, Federación Colombiana de Obstetricia y Ginecología; FIGO, Fédération Internationale de Gynécologie et d’Obstétrique; GOHO, Gottesfeld-Hohler Memorial Foundation; ICU, intensive care unit; IOG, Institute of Obstetricians and Gynaecologists; IS-PAS, International Society for Placenta Accreta Spectrum; MRI, magnetic resonance imaging; MSPE, Medical Student Performance Evaluation; NICE, National Institute for Health and Care Excellence; NICU, neonatal intensive care unit; NWIHP, National Women and Infants Health Programme; PAS, placenta accreta spectrum; PSGO, Polish Society of Gynecologists and Obstetricians; RANZCOG, Royal Australian and New Zealand College of Obstetricians and Gynaecologists; RCOG, Royal College of Obstetricians and Gynaecologists; SMFM, Society of Maternal-Fetal Medicine; SOGC, Society of Obstetricians and Gynaecologists of Canada; TAS, transabdominal ultrasonography; TV, transvaginal.

The epidemiology section demonstrated high agreement (75.0%) among guidelines providing recommendations on the topic (Figure 2; eTable 4 in Supplement 1). All guidelines recognized previous CD as a substantial risk factor for PAS, with risk increasing with the number of CDs. Consistently cited evidence includes a prospective, observational, multicenter cohort study by Silver et al,^28^ which reported PAS incidence increasing with the number of prior CDs (0.2%, 0.3%, 0.6%, 2.1%, 2.3%, and 6.7% after 1, 2, 3, 4, 5, and ≥6 CDs, respectively; for cases with placenta previa, rates increased to 3.3%, 11.1%, 40.0%, 61.0%, 67.0%, and 67.0%, respectively). A 2014 meta-analysis by Klar and Michels^29^ reported an odds ratio of 1.96 (95% CI, 1.41-2.74) for PAS after a prior CD. The incidence of PAS increased from 3.3% to 4.0% in patients with placenta previa with no previous CDs to 50.0% to 67.0% in patients with 3 or more prior CDs. In addition, the 2016 Nordic Obstetric Surveillance Study found that the odds ratio for PAS increased from 6.6 (95% CI, 4.4-9.8) after 1 CD to 17.4 (95% CI, 9.0-31.4) after 2 CDs and 55.9 (95% CI, 25.0-110.3) after 3 or more CDs.^30^

Other acknowledged risk factors included previous myomectomy, minor uterine operations (eg, curettage), and placenta previa or low-lying, although evidence supporting these associations was generally considered moderate to low. Additional factors, such as advanced maternal age, multiparity, multiple pregnancies, in vitro fertilization, anemia, smoking, and cocaine use, were inconsistently reported, reflecting variability in their recognition and evidence-based significance.

#### Prenatal and Antepartum Screening and Diagnosis

Prenatal screening showed 76.9% overall agreement, 7.7% disagreement, and 15.4% insufficient evidence (Figure 2; eTable 5 in Supplement 1). Ultrasonography, including color flow Doppler, is unanimously endorsed as the first-line screening modality for PAS. Four guidelines specifically highlight the utility of transvaginal ultrasonography for improved visualization of key structures.^11,12,14,24^ Most guidelines favor second-trimester screening, with 3 suggesting initiation in the first trimester.^14,23,24^ Patients with known risk factors, particularly a history of CD or placenta previa, are consistently identified as priority candidates; there is less agreement regarding anterior low-lying placenta cases. Standardized ultrasonographic descriptors are strongly encouraged to enhance diagnostic accuracy. Consistently cited evidence includes a 2013 meta-analysis by D’Antonio et al,^31^ which reported ultrasonographic sensitivity and specificity of 90.7% (95% CI, 87.2%-93.6%) and 96.9% (95% CI, 96.3%-97.5%), respectively. Individual ultrasonographic features showed high specificities (95%-100%) with varying sensitivities: placental lacunae, 77% (95% CI, 71%-83%); loss of retroplacental clear space, 66% (95% CI, 58%-74%); bladder border abnormalities, 50% (95% CI, 41%-58%); and color Doppler abnormalities, 91% (95% CI, 85%-95%).^31^ A 2016 systematic review by Jauniaux et al^32^ highlighted variations in key ultrasonographic findings by PAS severity. Common signs included loss of the clear zone (62.1% in accreta and 84.6% in increta), bridging vessels (71.4% in accreta), subplacental hypervascularity (60% in increta and 54.5% in percreta), and placental lacunae (82.4% in percreta). The role of MRI remains less definitive, with guidelines agreeing on its value in specific scenarios (eg, posterior placenta and inconclusive ultrasonography) but acknowledging conflicting evidence. Lastly, there is currently no recommended diagnostic biomarker screening for PAS.

### Antenatal Management and Required Expertise

#### Antenatal Management

Antenatal management exhibited 88.9% agreement and 11.1% insufficient evidence. No strong disagreement was identified (Figure 2; eTable 6 in Supplement 1). There is no unified guidance on the optimal surveillance timeframe for patients with suspected PAS. Guidelines generally agree that well-counseled patients with quick access to health care facilities may be managed in the outpatient setting if asymptomatic, whereas symptomatic patients, especially those with recurrent bleeding, should be admitted to the hospital. The recommended gestational age for inpatient admission varies, depending on clinical circumstances, the severity of symptoms, and individual risk factors. Early admission may be considered in cases with substantial symptoms or when there is concern for imminent preterm delivery. Corticosteroid administration is broadly recommended if there is vaginal bleeding or imminent risk of delivery before 35 weeks. One guideline extends this recommendation to 37 weeks, reflecting variability in practice.^12^ Recommendations for pharmacologic thromboprophylaxis vary substantially. Guidelines generally concur on the necessity of planned CD for patients with PAS. The optimal timing varies, with most recommending 34 to 35 weeks and some extending the window to 37 weeks.^11,14,15,19,21,25^

#### Expertise Required for Managing PAS Disorders

There was 100% agreement on the necessity of managing suspected PAS disorders in specialist centers (Figure 2; eTable 7 in Supplement 1). Key features required to define a specialist center include the following. First a multidisciplinary team should be available on site, including an experienced obstetrician, anesthesiologist, imaging expert, surgeon skilled in complex pelvic operations, urologist, neonatologist, interventional radiologist, and a physician specializing in hematology, hematopathology, and transfusion medicine pathology. Conflicting evidence exists concerning the need for colorectal and vascular surgeons. Although there is no clear definition of required expertise (eg, years of practice), most guidelines concur that an expert in PAS should possess substantial experience, specialized knowledge, and the necessary skills to effectively manage it. Second, structured discussions and individualized care plans, with birth planning and, where possible, a written protocol with surgical options, should be available. Third, substantial experience in managing PAS is needed, with protocols in place to guide care and manage imaging and blood results effectively. Fourth, capacity to perform a high-volume of complex pelvic operations is necessary. Fifth, on-site adult and neonatal critical care, blood product services, massive transfusion capabilities, and intraoperative blood salvage services are required. Sixth, continuous specialized training for operating room teams and maternal-fetal medicine specialists is needed.

#### Counseling

**Figure 3.**
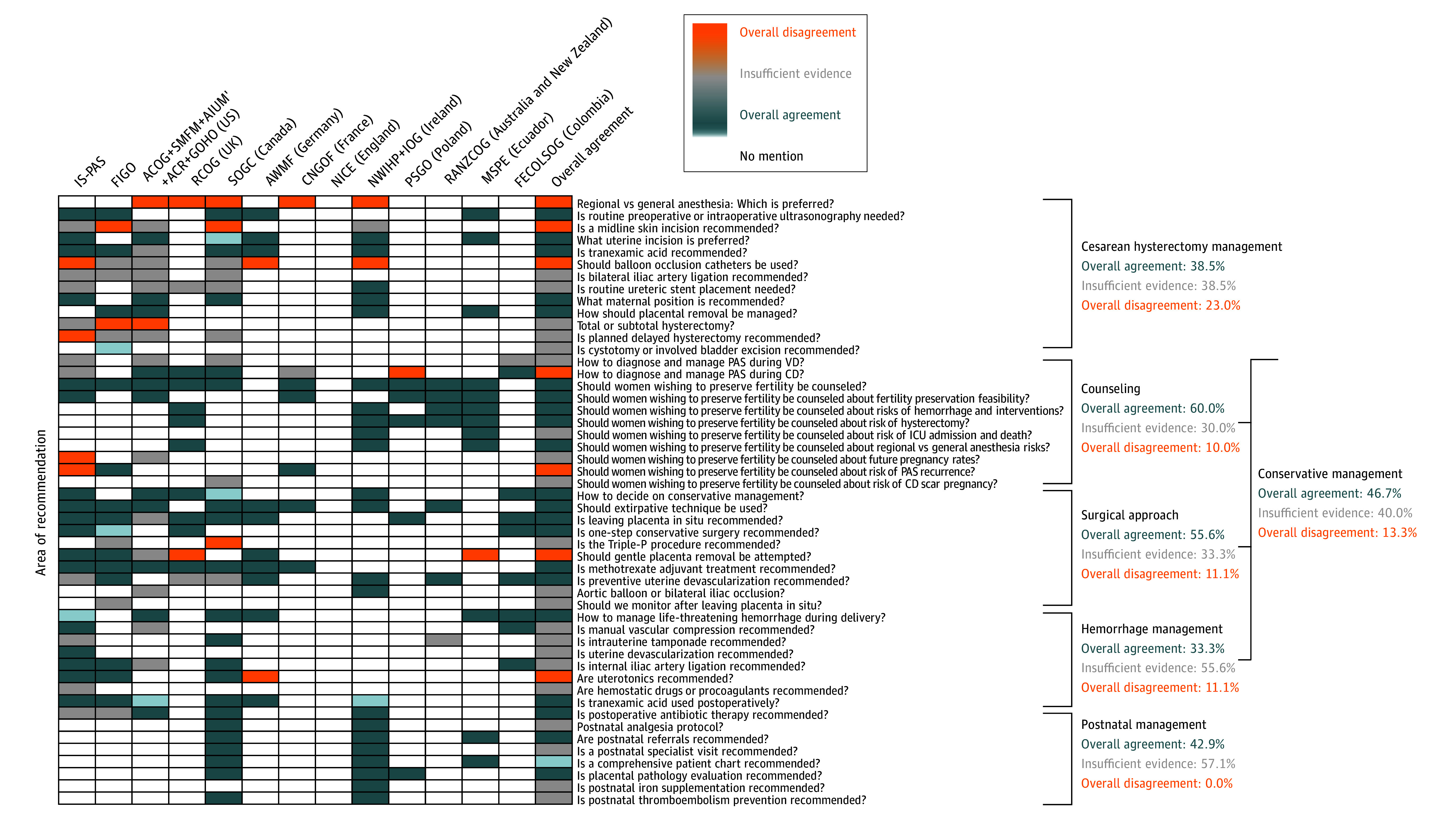
Surgical Treatment and Postnatal Management ACOG indicates American College of Obstetricians and Gynecologists; ACR, American College of Radiology; AIUM, American Institute of Ultrasound in Medicine; AWMF, Association of Scientific Medical Societies in Germany; CD, cesarean delivery; CNGOF, Collège National des Gynécologues et Obstétriciens Français; FECOLSOG, Federación Colombiana de Obstetricia y Ginecología; FIGO, Fédération Internationale de Gynécologie et d’Obstétrique; GOHO, Gottesfeld-Hohler Memorial Foundation; ICU, intensive care unit; IOG, Institute of Obstetricians and Gynaecologists; IS-PAS, International Society for Placenta Accreta Spectrum; MSPE, Medical Student Performance Evaluation; NICE, National Institute for Health and Care Excellence; NWIHP, National Women and Infants Health Programme; PAS, placenta accreta spectrum; PSGO, Polish Society of Gynecologists and Obstetricians; RANZCOG, Royal Australian and New Zealand College of Obstetricians and Gynaecologists; RCOG, Royal College of Obstetricians and Gynaecologists; SMFM, Society of Maternal-Fetal Medicine; SOGC, Society of Obstetricians and Gynaecologists of Canada; VD, vaginal delivery.

All guidelines strongly recommend comprehensive counseling for patients considering fertility preservation, ensuring they fully understand that although fertility preservation may be possible, it carries substantial risks, including massive hemorrhage, hysterectomy, and urinary tract damage (Figure 3; eTable 9 in Supplement 1). The uncertain benefits of conservative management should be clearly communicated. The extent and detail of discussions vary across guidelines, with some emphasizing the importance of addressing the feasibility of fertility preservation and risk of recurrence in future pregnancies. There is insufficient evidence to provide definitive guidance on future pregnancy rates and insufficient guidance on whether to mention the risks of intensive care unit admission, death, and future risk of cesarean scar pregnancies. All guidelines agree that the decision to pursue conservative management in suspected PAS should be made only after thorough counseling, prioritizing the woman’s preferences, and considered only in carefully selected cases in which necessary resources are available.

### Intrapartum Care

#### Cesarean Hysterectomy

The section on hysterectomy techniques showed poor consensus (38.5% [5 of 13] agreement, 38.5% [5 of 13] insufficient evidence, and 23.0% [3 of 13] disagreement) (Figure 3; eTable 8 in Supplement 1). Consensus exists on using preoperative or intraoperative ultrasonography to map placental edges, using the modified dorsal lithotomy position for delivery, selecting the uterine incision site based on antenatal imaging and intraoperative findings to ensure a sufficient distance from the placenta, avoiding placental removal attempts to prevent bleeding, and administering tranexamic acid immediately before or during CD to manage hemorrhage. However, substantial disagreement and evidence gaps persist in key areas, including anesthesia choice, with some guidelines favoring general anesthesia^12^ and others recommending regional techniques,^14^ ultimately advising a case-by-case approach based on patient preference and safety^11,15,25^; and skin incision technique, with some guidelines supporting a midline incision despite weak evidence, especially when the placental superior margin extends beyond the lower uterine segment,^10,11,14,15^ and others emphasizing the safety and satisfaction of a large transverse incision, noting the lack of evidence comparing outcomes between techniques.^13^

#### Conservative Management

In the main area of conservative management, 14 of 30 topics (46.7%) demonstrated agreement among guidelines, 4 of 13 (13.3%) demonstrated disagreement, and 12 of 30 (40.0%) had insufficient evidence (fertility counseling: 60.0% [6 of 10] agreement, 30.0% [3 of 10] insufficient evidence, and 10.0% [1 of 10] disagreement; surgical approach: 55.6% [5 of 9] agreement, 33.3% [3 of 9] insufficient evidence, and 11.1% [1 of 9] disagreement) (Figure 3; eTable 9 in Supplement 1). All guidelines unanimously discourage forcible manual placenta removal, whereas consensus on gentle attempted removal is mixed, with some guidelines considering it acceptable in cases of suspected false-positive results with no clear signs of PAS^13,22,24^ and others advising against it.^15,21^

The leaving the placenta in situ approach is generally accepted in carefully selected cases, provided there is detailed patient counseling, access to expert teams, and close monitoring. However, guidelines lack detailed monitoring protocols. The one-step conservative surgery approach is considered feasible for focal central disease, particularly with less than 50.0% invasion of the anterior uterine surface. The triple-P procedure has limited evidence regarding efficacy, and its feasibility is supported by only one guideline.^14^ Adjuvant methotrexate therapy and preventive surgical and radiologic uterine devascularization are generally not recommended due to insufficient evidence and potential harm.

#### Hemorrhage Management

Guidelines consistently emphasize the importance of timely interventions for life-threatening hemorrhage (Figure 3; eTable 9 in Supplement 1). Despite this, 5 of 9 topics (55.6%) in the area of hemorrhage management demonstrated insufficient evidence. Although hysterectomy is universally recognized as the definitive treatment for massive hemorrhage in unstable patients, evidence for interventions such as manual vascular compression (eg, aorta and common iliacs), intrauterine tamponade, uterine devascularization, and internal iliac artery ligation, is weaker. Preoperative or intraoperative aortic or iliac balloon occlusion catheter use lacks consensus, with some guidelines accepting it and others citing insufficient evidence. Similarly, bilateral internal iliac artery ligation and ureteric stent placement remain unsupported by strong evidence.

High agreement exists on using tranexamic acid to manage hemorrhage, supported by high-quality evidence, with particular interest in its application for treating postpartum hemorrhage in LMICs.^33,34^ However, its specific role in PAS management remains unclear because no data are currently available. The use of uterotonic agents is debated, with some guidelines recommending them and others advising against their use unless there is no clinical evidence of PAS.

### Postnatal Care

In the area of postnatal care, 4 of 7 topics (57.1%) had high levels of insufficient evidence, with only 4 guidelines addressing key aspects (Figure 3; eTable 10 in Supplement 1).^11,14,21,27^ Among these, there is consensus on the importance of comprehensive patient records, including detailed intraoperative findings and placental pathologic test results. Guidelines also strongly support postnatal referrals, particularly for social work services and perinatal mental health, emphasizing the need for ongoing care beyond the immediate postpartum period.

Postoperative antibiotic therapy is widely recommended, especially when conservative measures such as pelvic tamponade or leaving the placenta in situ are used, but evidence for efficacy is lacking. There is insufficient evidence on postnatal analgesia protocols tailored to PAS-related interventions. Despite the high risk of anemia, consensus is lacking on iron supplementation strategies. Lastly, recommendations for thromboembolism prevention varies, with some guidelines favoring pharmacologic interventions, such as low-molecular-weight heparin, and others advocating for nonpharmacologic measures, such as compression stockings and early mobilization.

## Discussion

### Areas of Consensus

Our review found several areas of strong agreement among national and international professional societies. All guidelines recognize previous CD as a major risk factor and emphasize incorporating PAS risk into mode of delivery counseling to support informed decision-making.^12,14,15,18,19,21,24,25^ Ultrasonography and color flow Doppler are unanimously endorsed as the primary screening tools, valued for their accuracy, accessibility, and cost-effectiveness when performed by skilled operators. There is also 100% agreement on the need for care of patients with PAS in specialist centers with appropriate expertise and resources.

Guidelines generally support planned CD for patients with PAS. We acknowledge the possibility of vaginal delivery in specific scenarios, such as when the placenta is positioned high and the patient opts for conservative management. For cesarean hysterectomy, there is high agreement on using the modified dorsal lithotomy position and preoperative or intraoperative ultrasonography to map placental edges, particularly in high-resource settings. High agreement exists on the possible role of tranexamic acid for managing massive hemorrhage, particularly in LMICs, although evidence specific to tranexamic acid use in PAS remains unavailable.

### Areas of Disagreement and Discrepancies

Despite areas of consensus, significant variability exists in recommendations for complex aspects of PAS management, such as antenatal management, conservative approaches, and hemorrhage control techniques. Although most guidelines favor second-trimester screening, some suggest starting to screen in the first trimester. Recommendations for the timing of delivery range from 34 to 35 to 37 weeks, suggesting the need for prospective studies, including cervical length measurements to refine timing recommendations.

Variations in recommendations for general vs regional anesthesia reflect the current need for case-by-case decision-making based on patient factors and wishes as well as local expertise. No CPGs specifically focused on obstetric anesthesia were included in this review. Disagreements exist regarding the appropriateness of midline vs large transverse skin incision during hysterectomy. Some guidelines support a midline skin incision despite low and weak evidence, particularly if the placental superior margin is outside the lower uterine segment, whereas others report no evidence of benefit and favor a large transverse incision based on long-term safety and patient satisfaction data.

### Areas With Limited Guidance

This review identified several critical gaps in PAS management guidance. Inconsistent reporting of minor risk factors suggests a lack of robust evidence and the need for further research to establish a clearer consensus. Recommendations on magnetic resonance imaging use vary widely in terms of timing, application, and use of contrast media. The use of diagnostic biomarkers also remains uncertain because current evidence does not support their routine use. Serial growth scans are not recommended due to the absence of a proven association between PAS and fetal growth restriction.^35,36^

Protocols for monitoring and managing complications in conservative treatment approaches remain insufficient. Paucity of data exists on interventions for massive hemorrhage, with techniques such as manual vascular compression, tamponade, devascularization, artery ligation, and hemostatic or procoagulant agents, lacking validation. Postoperative care guidance is also limited, particularly regarding analgesia protocols, iron supplementation, and thromboembolism prevention. Current guidelines fail to address long-term follow-up, including recommendations for future pregnancies and health monitoring. There is little focus on the psychological impact of PAS on patients and families, despite the profound physical and emotional toll of such a challenging diagnosis and potentially traumatic birth.^37,38^ Multidisciplinary care emphasizing mental health and patient-centered decision-making could help address these unmet needs and offer more holistic care.^39^

A critical gap is the absence of tailored recommendations for LMICs, where limited resources and infrastructure often hinder adherence to established guidelines. A recent study highlighted deviations in PAS care in middle-income countries due to these constraints.^40^ The importance of this issue is magnified when considering 2023 data from the United Nations Revision of World Population Prospects, which indicates that 90% of global births occur in LMICs (71% and 19% in middle- and low-income nations, respectively),^41^ and the increasing trend in CD rates worldwide, particularly in LMICs in Eastern and Southern Asia and Northern Africa.^42^ Tailored international guidelines and training programs for low-resource settings are urgently needed to improve outcomes for patients at risk of PAS worldwide.

### Strengths and Limitations

This review’s strengths include its comprehensive nature, covering guidelines from multiple national and international societies, and its systematic approach without language restrictions to extract and evaluate recommendations. The involvement of a panel of PAS guideline developers ensured high accuracy of assessments and results.

However, this study also has limitations that should be acknowledged. The review focused solely on published guidelines, which may not capture all existing clinical practices across different regions and health care systems. Practices may vary widely by country, continent, or even individual centers due to resource availability and/or culture. Additionally, the dynamic nature of PAS research means some guidelines may not incorporate the latest evidence.

The review emphasized guideline content comparison rather than evaluating the quality of their development processes. Such an assessment was previously conducted by Capannolo et al^4^ using the Appraisal of Guidelines for Research and Evaluation (AGREE II) tool on 6 of the 14 guidelines we included, finding most to be high quality (scores >60.0%), except for 2 scoring 57.0%.

## Conclusions

In this systematic review of PAS guidelines, we identified substantial areas of consensus, particularly in risk factor identification and the necessity for specialized care. However, discrepancies and evidence gaps persist in crucial areas, such as conservative management, hemorrhage control, and postnatal care, emphasizing the urgent need for high-quality prospective research to fill these gaps and standardize care protocols. The lack of guidelines tailored to LMICs highlights a critical need for context-specific recommendations that consider resource limitations. With PAS incidence increasing worldwide, prioritizing these research areas and developing comprehensive, globally applicable guidelines are essential steps toward improving outcomes for all patients affected by this life-threatening condition.

## Notes

Supplement 1. **eTable 1**. Professional characteristics of experts in round 1 of the survey **eTable 2**. Expert consensus on controversial issues: Round 1 survey results **eTable 3.** Professional characteristics of experts in round 2 of the survey **eTable 4**. Summary and comparative analysis of guideline recommendations on the epidemiology of PAS disorders **eTable 5.** Summary and comparative analysis of guideline recommendations on prenatal screening and diagnosis of PAS disorders **eTable 6.** Summary and comparative analysis of guideline recommendations on the antenatal management of PAS disorders **eTable 7**. Summary and comparative analysis of guideline recommendations on expertise required for managing PAS disorders **eTable 8.** Summary and comparative analysis of guideline recommendations on cesarean hysterectomy management of PAS disorders **eTable 9.** Summary and comparative analysis of guideline recommendations on the conservative management of PAS disorders for women wishing fertility preservation **eTable 10.** Summary and comparative analysis of guideline recommendations on postnatal management of PAS disorders

Supplement 2.
Data Sharing Statement
