# Supplementary material for: Guidelines on Placenta Accreta Spectrum Disorders: A Systematic Review
Source: JAMA Netw Open. 2025 Jul 18;8(7):e2521909. doi: 10.1001/jamanetworkopen.2025.21909 (PMC12274978; doi:10.1001/jamanetworkopen.2025.21909)
Supplement: Supplement 1. — eTable 1. Professional characteristics of experts in round 1 of the survey eTable 2. Expert consensus on controversial issues: Round 1 survey results eTable 3. Professional characteristics of experts in round 2 of the survey eTable 4. Summary and comparative analysis of guideline recommendations on the epidemiology of PAS disorders eTable 5. Summary and comparative analysis of guideline recommendations on prenatal screening and diagnosis of PAS disorders eTable 6. Summary and comparative analysis of guideline recommendations on the antenatal management of PAS disorders eTable 7. Summary and comparative analysis of guideline recommendations on expertise required for managing PAS disorders eTable 8. Summary and comparative analysis of guideline recommendations on cesarean hysterectomy management of PAS disorders eTable 9. Summary and comparative analysis of guideline recommendations on the conservative management of PAS disorders for women wishing fertility preservation eTable 10. Summary and comparative analysis of guideline recommendations on postnatal management of PAS disorders [file jamanetwopen-e2521909-s001.pdf]

Supplemental Online Content

Bonanni G, Lopez-Giron MC, Allen L, et al. Guidelines on placenta accreta spectrum disorders: a systematic review. *JAMA Netw Open*. 2025;8(7):e2521909. doi:10.1001/jamanetworkopen.2025.21909

eTable 1. Professional characteristics of experts in round 1 of the survey

eTable 2. Expert consensus on controversial issues: Round 1 survey results

eTable 3. Professional characteristics of experts in round 2 of the survey

eTable 4. Summary and comparative analysis of guideline recommendations on the epidemiology of PAS disorders

eTable 5. Summary and comparative analysis of guideline recommendations on prenatal screening and diagnosis of PAS disorders

eTable 6. Summary and comparative analysis of guideline recommendations on the antenatal management of PAS disorders

eTable 7. Summary and comparative analysis of guideline recommendations on expertise required for managing PAS disorders

eTable 8. Summary and comparative analysis of guideline recommendations on cesarean hysterectomy management of PAS disorders. The reported level of evidence is indicated in *italics*.

eTable 9. Summary and comparative analysis of guideline recommendations on the conservative management of PAS disorders for women wishing fertility preservation

eTable 10. Summary and comparative analysis of guideline recommendations on postnatal management of PAS disorders

This supplemental material has been provided by the authors to give readers additional information about their work.

© 2025 Bonanni G et al. *JAMA Network Open.*

eTable 1. Professional characteristics of experts in round 1 of the survey

PAS, Placenta Accreta Spectrum; IQR, Interquartile Range

**Characteristics of experts**	N = 15
**Country**, *n (%)*	
USA	6 (40)
UK	2 (13.3)
Canada	2 (13.3)
France	1 (6.7)
Portugal	1 (6.7)
Belgium	1 (6.7)
Colombia	1 (6.7)
Ireland	1 (6.7)
**Years of experience in PAS,** *median (IQR)*	15 (12-25)
≤10	3 (20)
11 to 20	7 (46.7)
>20	5 (33.3)
**Approximate N of PAS evaluated per year at expert's institute,** *median (IQR)*	20 (15 - 50)
≤15	6 (40)
16 - 30	5 (33.3)
>30	4 (26.7)

eTable 2. Expert consensus on controversial issues: Round 1 survey results

**Response**	**Q1 Minor surgical procedures**	**Q2 Other risk factors**	**Q3 Timing of Ultrasound screening**	**Q4 Colorectal and vascular surgeon**	**Q5 Ureteric stent**	**Q6 Total vs subtotal hysterectomy**	**Q7 Planned delayed hysterectomy**	**Q8 VD intrapartum diagnosis**
Good agreement	9 (64.3)	1 (7.1)	7 (50)	3 (21.4)	4 (28.6)	0 (0)	0 (0)	2 (14.3)
Considerable disagreement	2 (14.3)	3 (21.43)	6 (42.9)	3 (21.4)	4 (28.6)	3 (21.4)	6 (42.9)	3 (21.4)
Insufficient evidence	3 (21.4)	10 (71.4)	1 (7.1)	8 (57.1)	6 (42.9)	11 (78.6)	8 (57.1)	9 (64.3)

**Response**	**Q9 CD intrapartum diagnosis**	**Q10 Counseling: major risks**	**Q11 Triple-P Procedure**	**Q12 Manual vasc. Compression**	**Q13 Iliac a. ligation**	**Q14 Postnatal referrals**	**Q15 Patient chart**	**Q16 Placenta pathology**
Good agreement	4 (28.6)	4 (28.6)	0 (0)	9 (64.3)	5 (35.7)	3 (21.4)	8 (57.1)	9 (64.3)

© 2025 Bonanni G et al. *JAMA Network Open.*

Considerable disagreement	7 (50)	1 (7.1)	2 (14.3)	0 (0)	2 (14.3)	2 (14.3)	0 (0)	0 (0)
Insufficient evidence	2 (14.3)	9 (64.3)	12 (85.7)	5 (35.7)	7 (50)	9 (64.3)	6 (42.9)	5 (35.7)

eTable 3. Professional characteristics of experts in round 2 of the survey

**Characteristics of experts**	N = 15
**Country**, *n (%)*	
USA	5 (33.3)
Canada	3 (20)
UK	2 (13.3)
France	1 (6.7)
Poland	1 (6.7)
Belgium	1 (6.7)
Colombia	1 (6.7)
Ireland	1 (6.7)
**Years of experience in PAS,** *median (IQR)*	15 (12-25)
≤10	3 (20)
11 to 20	8 (53.3)
>20	4 (26.7)
**Approximate N of PAS evaluated per year at expert's institute,** *median (IQR)*	20 (15 - 50)
≤15	8 (53.3)
16 - 30	5 (33.3)
>30	2 (13.3)

© 2025 Bonanni G et al. *JAMA Network Open.*

eTable 4. Summary and comparative analysis of guideline recommendations on the epidemiology of PAS disorders. The reported level of evidence is indicated in *italics*.

Topic	FIGO (2018)	ACOG+ SMFM (USA, 2021)	RCOG (UK, 2018)	SOGC (Canada, 2019)	AWMF (Germany, 2022)	CNGOF (France, 2016)	MSPE (Ecuador, 2017)	FECOLSOG (Colombia, 2023)	Agreement[a]
**Risk factor: previous cesarean delivery**	Yes *High and strong* (risk rises with #CDs)	Yes	Yes *Moderate* (risk rises with #CDs)	Yes *High and strong* (risk rises with #CDs)	Yes *High and strong* (risk rises with #CDs)	Yes *High and strong*	Yes *Weak* (especially within 12 mos.; risk rises with #CDs)	Yes *High and strong*	✔
**Risk factor: myomectomy (scar)**	Yes *Low and weak*		Yes *Moderate*	Yes *Weak*	Yes (“transmural”) *High and strong*	Yes *Moderate*	Yes *Weak*	Yes	✔
**Risk factor: minor surgical procedures (e.g., uterine curettage)**	Yes *Low and weak*	Yes (prior surgeries or curettage)	Yes (“repeated curettage”) *Moderate*	Yes	Unclear (“other uterine surgeries”)	Unclear (“history of uterine surgeries”)	Yes *Weak*		✔
**Risk factor: history of PAS**	Yes		Yes *Moderate*		Yes (“placental implantation disorders”)	Yes *Moderate*			✔
**Risk factor: Placenta previa or low-lying**	Yes *High and strong* (especially with CD history)	Yes	Yes (especially if associated with history of CD) *Conflicting*	Yes	Yes	Yes *Moderate*	Yes (manage as PAS) *Weak*	Yes (active search required) *High and strong*	✔
**Other risk factors**		Maternal age, multiparity, Asherman’s, abnormal biomakers		Maternal age, IVF, fibroid embolization (*weak*), intrauterine adhesions (*weak*), congenital uterine abnormality, history of pelvic irradiation		Maternal age, IVF	Maternal age 35+, multiparity, multiple pregnancies, anemia, smoking, cocaine usage		⚠
**Preconception: counseling and prevention**							Good practice point. Should cover all PAS risk factors, especially smoking.		⚠
**Standardization of protocols and terminology for clinical and histopathological diagnosis of PAS**	Recommended; to obtain new and more accurate data *High and strong*		Good practice point						⚠

[a] Areas with good agreement, considerable disagreement, or insufficient guidance are flagged by a green checkmark, red x, or a black triangle with the exclamation mark, respectively.
PAS, Placenta Accreta Spectrum; FIGO, Fédération Internationale de Gynécologie et d'Obstétrique; RCOG, Royal College of Obstetricians and Gynaecologists; UK, United Kingdom; SOGC, Society of Obstetricians and Gynaecologists of Canada; AWMF, Association of Scientific Medical Societies in Germany; CNGOF, Collège National des Gynécologues et Obstétriciens Français; MSPE, Ministerio de Salud Publica del Ecuador; FECOLSOG, Federación Colombiana de Obstetricia y Ginecología; CD, Cesarean Delivery; IVF, In Vitro Fertilization; LBW, Low Birth Weight

© 2025 Bonanni G et al. *JAMA Network Open.*

eTable 5. Summary and comparative analysis of guideline recommendations on prenatal screening and diagnosis of PAS disorders. The reported level of evidence is indicated in *italics*.

Topic	FIGO (2018)	ACOG + SMFM+ AIUM + ACR + GOHO (USA, 2021)	RCOG (UK, 2018)	SOGC (Canada, 2019)	AWMF (Germany, 2022)	CNGOF (France, 2016)	NICE (UK, 2024)	NWIHP + IOG (Ireland, 2022)	PSGO (Poland, 2018)	MSPE (Ecuador, 2017)	RANZCOG (Australia and New Zeland, 2023)	Agreement[a]
**Role of antenatal screening**		Helps in optimizing management and outcomes	Helps in planning care; lowers maternal morbidity and mortality *Conflicting*	Helps in planning definite management and optimizing outcomes	Helps in implementing measures to reduce maternal morbidity and mortality *High and strong*	Helps in care improvement *Moderate*						✓
**US as first line screening modality**	Recommended (cost-effective and readily accessible) *High and strong*	Recommended *High and strong*	Recommended (highly accurate with expert operator) *Weak*	Recommended	Recommended *High and strong*	Recommended (combination of 2D and color Doppler) *Moderate*	Recommended *High and strong*	Recommended *High and strong*	Recommended ("basic examination")	Recommended *High and strong*	Recommended (accurate, available, affordable)	✓
**US screening: TAS vs TVUS**		TAS + TVUS recommended *High and strong*		TVUS recommended	TVUS recommended			TVUS more clearly identifies some structures				✓
**US screening: timing**	2nd trimester	From early first trimester onwards, throughout pregnancy		1st and 2nd trimester (including CS scar pregnancy screening)	1st and 2nd trimesters *Strong*		Around 28 wks, no later than 29 wks	2nd trimester (placental location clearly documented)				✓
**US Screening: Population**	Women with history of CD, especially if placenta is anterior, low lying or previa *Medium and strong*	Women with risk factors, especially prior CD and previa	Women with history of CD and placenta anterior low-lying or previa *Conflicting*	Women with risk factors *Moderate*	Women with anamnestic (previous surgery) or diagnostic (placenta previa) risks		Women with history of CD and placenta low-lying or previa	Women with history of CD and placenta previa *Expert opinion*				✓
**US screening: standardized**	Recommended *Medium and strong*	Recommended *Expert opinion*		Recommended				Recommended[b] *High and strong*			Recommended (consider using a template)	✓

© 2025 Bonanni G et al. *JAMA Network Open.*

**descriptions**												
**US screening: limitations**	Operator-dependent *High and strong*	Cannot exclude PAS; operator-dependent and inter-observer variation; bias in patients' selection; posterior placenta, high BMI and uterine leiomyomata can decrease sensitivity *Moderate*		Operator-dependent;influenced by GA and bladder filling			Cannot exclude PAS; operator-dependend	Cannot completely exclude PAS *High and strong*				✓
**US screening: Doppler**		Recommended after 10 weeks		Recommended		Recommended *Moderate*	Recommended	Recommended	Recommended	Recommended to confirm diagnosis[c] *Moderate*		✓
**MRI role**	Not essential; helpful to check pelvic invasion *Medium and weak*	Unclear *Insufficient*	May be helpful to check pelvic invasion, especially with posterior placenta *Weak*	May be helpful *Conflicting*	Not routine; may be useful in unclear cases *Expert opinion*	Helpful for diagnosis for posterior placenta of doubtful cases *Moderate*	May be helpful to check pelvic invasion, especially with posterior placenta	May be helpful	Adds extra details for posterior placenta cases	Reserved for uncertain or inconclusive cases *Expert opinion*	May be helpful in uncertain cases or with posterior placenta	✓
**MRI: timing**				<34 wks						24-30 wks		⚠
**MRI: use of contrast media**			*Insufficient*	Not recommended	Not routinely recommended *Expert opinion*							✗
**Criteria for expert referral**	Previous CD + one between placenta previa or anterior low lying placenta (<2 cm from internal OS after 16 wks)	If any suspicion arises or any risk factors	Any US feature suggestive of PAS *Moderate*	Clinical risk factors and anterior placenta previa *Moderate*	If any suspicion arises	If any suspicion arises *Expert opinion*	Clinical risk factors and low-lying placenta/ previa	If any suspicion arises *Moderate*		Risk factors or if any suspicion arises *Expert opinion*	If any suspicion arises *Expert opinion*	✓
**Diagnostic biomarkers use**	Insufficient on their benefit			Increased 1st trimester PAPP-A	Limited to the scope of							✗

© 2025 Bonanni G et al. *JAMA Network Open.*

Insufficient to recommend use	registered clinical studies *Conflicting*

[a] Areas with good agreement, considerable disagreement, or insufficient guidance are flagged by a green checkmark, red x, or a black triangle with the exclamation mark, respectively.
[b] Diagnostic features: loss of clear zone; myometrial thinning; uterine bulge, focal exophytic placental mass; through serosa, parametrium; bladder wall interruption; lacunae; Doppler: placenta lacunae feeder vessels; abnormal vasculature at placental-myometrial interface, serosa, bladder
[c] Diagnostic features: turbulent flow with abnormal areas of hypervascularity with dilated blood vessels at the myometrial interface

PAS, Placenta Accreta Spectrum; FIGO, Fédération Internationale de Gynécologie et d'Obstétrique; ACOG, American College of Obstetricians and Gynaecologists; SMFM, Society of Maternal Fetal Medicine; AIUM, American Institute of Ultrasound in Medicine; ACR, American College of Radiologists; GOHO, Gottesfeld Hohler Memorial Society; RCOG, Royal College of Obstetricians and Gynaecologists; UK, United Kingdom; SOGC, Society of Obstetricians and Gynaecologists of Canada; AWMF, Association of Scientific Medical Societies in Germany; CNGOF, Collège National des Gynécologues et Obstétriciens Français; NICE, National Institute for Health and Care Excellence; NWIHP, National Women and Infants Health Programme's; IOG, Institute of Obstetricians and Gynaecologists; PSGO, Polish Society of Gynecologists and Obstetricians; MSPE, Ministerio de Salud Publica del Ecuador; RANZCOG, Royal Australian and New Zealand College of Obstetricians and Gynaecologists; CD, Cesarean Delivery; US, ultrasound; TAS, Trans-abdominal sonography; TVUS, Trans-vaginal sonography; MRI, Magnetic Resonance Imaging; wks, weeks of gestation; VD, Vaginal Delivery; GA, Gestational Age; PAPP-A, serum Pregnancy-Associated Plasma Protein-A

eTable 6. Summary and comparative analysis of guideline recommendations on the antenatal management of PAS disorders. The reported level of evidence is indicated in *italics*.

Topic	IS-PAS (2019)	FIGO (2018)	ACOG + SMFM (USA, 2021)	RCOG (UK, 2018)	SOGC (Canada, 2019)	CNGOF (France, 2016)	NICE (UK, 2024)	NWIHP + IOG (Ireland, 2022)	PSGO (Poland, 2018)	RANZCOG (Australia and New Zeland, 2023)	MSPE (Ecuador, 2017)	FECOLSOG (Colombia, 2023)	Agreement[a]
**Surveillance timeframe**			US scan for placental location, previa and bladder invasion assessmenta t 18-20, 28-30, 32-34 wks *Reasonable*		US scan for fetal growth assessment during serial visits			US scan for fetal growth assessment at 28, 32, and 34 wks *Expert opinion*					⚠
**Expectant outpatient management vs antenatal admission**	Outpatient feasible for asymptomatic, counseled patients with fast hospital access. *Weak/Conflicting*		Antenatal admission if antepartum bleeding		Admission based on symptoms and/or geography. *Expert opinion*			Case-by-case decision based on symptoms, distance from hospital, social factors *Expert opinion*					✔
**Symptomatic women hospital admission**	Recommended *Weak*		Recommended		Recommended			Recommended (until delivery if recurrent bleeding, otherwise for					✔

© 2025 Bonanni G et al. *JAMA Network Open.*

								a period of observation)					
**Symptomatic women management**	According to local protocols and expertise *Conflicting*			According to contingency plan previously developed *Expert opinion*	Advise women to stay near regional care and avoid remote or foreign locations. Provide diagnosis and care location documentation for ambulance transfers.						Delivery if active bleeding regardless of GA		✓
**Planned delivery**	Recommended	Recommended (all resource settings) *High and strong*	Recommended *Expert opinion*	Recommended	Recommended *High and strong*	Recommended	Recommended	Recommended	Recommended *Conflicting*	Recommended *Very low*	Recommended	Recommended	✓
**Planned delivery: Time of delivery**	Individualized. 36 wks if asymptomatic and with no risk of preterm birth, otherwise 34 wks. *Conflicting*	34-35 wks	34^{+0}-35^{+6} wks (earlier if symptoms) *Strong*	35^{+0}-36^{+6} wks *Expert opinion*	34^{+0}-36^{+6} wks (earlier if symptoms)	34^{+0}-37^{+6} wks (case-by-case decision based on risk factors)		34^{+0}-36^{+6} wks (prior to 34 wks in selected cases)	34-37 wks	Individualized. *Expert opinion*	35-36 wks (upon newborn team consultation)	34^{+0}-36^{+6} wks (asymptomatic)	✓
**Planned delivery: Mode of delivery**			Planned CD		Elective CD			Elective CD *Moderate*	Elective CD *Conflicting evidenc*		Elective CD *Conflicting*		✓
**Planned delivery: corticosteroids administration timing**	Individualized approach, per local protocol *Conflicting*		Recommended if <37 wks		Recommended if vaginal bleeding and/or imminent risk of delivery <35 wks			Recommended if <35 wks			Recommended if risk of preterm birth within 24-34 wks		✓
**Pharmacological thromboprophylaxis**					LMWH (4-6 wks post-op), based on individual risk assessment and local protocols			Based on individual risk assessment, risk of bleeding and timing of birth *Moderate*			Based on individual risk assessment *Conflicting*		⚠

[a]Areas with Areas with good agreement, considerable disagreement, or insufficient guidance are flagged by a green checkmark, red x, or a black triangle with the exclamation mark, respectively.

© 2025 Bonanni G et al. *JAMA Network Open.*

PAS, Placenta Accreta Spectrum; IS-PAS, International Society - Placenta Accreta Spectrum; FIGO, Fédération Internationale de Gynécologie et d'Obstétrique; ACOG, American College of Obstetricians and Gynaecologists; SMFM, Society of Maternal Fetal Medicine; RCOG, Royal College of Obstetricians and Gynaecologists; UK, United Kingdom; SOGC, Society of Obstetricians and Gynaecologists of Canada; CNGOF= Collège national des gynécologues et obstétriciens; ACR, American College of Radiologists; NICE, National Institute for Health and Care Excellence; NWIHP, National Women and Infants Health Programme's; IOG, Institute of Obstetricians and Gynaecologists; PSGO, Polish Society of Gynecologists and Obstetricians; RANZCOG, Royal Australian and New Zealand College of Obstetricians and Gynaecologists; MSPE, Ministerio de Salud Publica del Ecuador; FECOLSOG, Federación Colombiana de Obstetricia y Ginecología; CD, Cesarean Delivery; wks, weeks of gestation; GA, Gestational Age; TVU, transvaginal ultrasound; LMWH,

© 2025 Bonanni G et al. *JAMA Network Open.*

eTable 7. Summary and comparative analysis of guideline recommendations on expertise required for managing PAS disorders. The reported level of evidence is indicated in *italics*

Topic	IS-PAS (2019)	FIGO (2018)	ACOG + SMFM (USA, 2021)	RCOG (UK, 2018)	SOGC (Canada, 2019)	AWMF (Germany, 2022)	CNGOF (France, 2016)	NICE (UK, 2024)	NWIHP + IOG (Ireland, 2022)	RANZCOG (Australia and New Zeland, 2023)	MSPE (Ecuador, 2017)	FECOLSOG (Colombia, 2023)	Agreement[a]
**Referral after diagnosis and delivery location**		Center of excellence<br>*High and strong*	Experienced level III or IV center<br>*High and strong*	Specialist center	Regional dedicated center<br>*High and strong*	Structure with the greatest possible expertise<br>*High and strong*		Specialist maternity service	Specialist center<br>*Moderate*	*Conflicting*	Third level center in case of absent multidisciplinary team in the hospital unit	Regionally defined referral center<br>*High and strong*	✓
**Time of referral**						Early prenatal stage. In case of intraoperative diagnosis and clinical stability, emergency transfer should be considered.		After specialist US confirming suspicion (by 29 wks)<br>*Expert opinion*	By 24 wks. If diagnosed after 24 wks, within 7 working days.	Immediately after diagnosis or if high suspicion	24 wks in case of high risk of bleeding<br>*Weak*	Immediately after diagnosis, no minimum GA	✓
						**Conditions/features defining a "specialist center for PAS"**							
**Team availability**	Necessary	Necessary	Necessary		Necessary	Necessary<br>*Strong*			Necessary				✓
**Case discussion and individual care plan**	Necessary			Necessary (with birth planning; elective CD if possible)	Necessary (protocol-based care recommended)	Necessary<br>*Strong*		Necessary (protocol-based)	Necessary (discussion of patient's history, review of imagin and blood results)	Necessary	Necessary	Necessary (written protocol with surgical options)	✓
**Significant experience in managing PAS**	Necessary	Necessary			Necessary<br>*Expert opinion*			Necessary				Necessary	✓

© 2025 Bonanni G et al. *JAMA Network Open.*

**Surgical capacity**	Necessary	Necessary *Moderate*	Necessary		Necessary *Expert opinion*		Necessary *Expert opinion*					Necessary *High and strong*	✓
**Staff training**			Necessary in III level or higher center		Recommended (OR team and MFM/MIS fellow)				Recommended (theatre staff) *High and strong*			Recommended (online and not, for all interested professionals) *High and strong*	✓
**Adult ICU on site**	Necessary *High and strong*	Necessary *Moderate and strong*		Necessary *Conflicting*	Recommended	Necessary *Strong*	Necessary *Expert opinion*	Necessary *High and strong*		Necessary	Necessary (availability should be verified before delivery) *High and strong*		✓
**NICU on site**	Necessary *High and strong*	Necessary *Moderate and strong*		Necessary *Conflicting*	Recommended	Necessary *Strong*		Necessary *High and strong*	Recommended	Recommended (or special care 32+ wks nursery)	Necessary		✓
**Blood product access**	Necessary *High and strong*	Necessary	Necessary	Necessary	Necessary	Necessary *Strong*	Necessary (rapid availability should be verified) *Expert opinion*	Necessary (rapid availability of cross-matched blood should be verified) *High and strong*	Necessary (cross-matched blood)	Necessary	Necessary		✓
**Massive transfusion facilities**	Necessary *High and strong*		Necessary *Expert opinion*		Necessary	Necessary			Necessary	Necessary for conservative management			✓
**Intraoperative blood salvage service available**	Necessary *High and strong*	Recommended when available (high-resource settings) *Weak*	Could be considered when available		Necessary			Could be considered	Recommended	Could be considered			✓
**Multidisciplinary team**													

© 2025 Bonanni G et al. *JAMA Network Open.*

**MDT**	Necessary *High and strong*	Necessary *High and strong*	Necessary *High and strong*	Necessary *High and strong*	Necessary *High and strong*	Necessary *High and strong*	Necessary	Necessary *High and strong*	Necessary *High and strong*	Necessary	Necessary	Necessary *High and strong*	✓
**Imaging expert**	Necessary *High and strong*		Recommended		Necessary				Necessary	Necessary			✓
**Experienced obstetrician**	Necessary		Recommended	Necessary (the most senior available) *Expert opinion*	Necessary	Recommended		Necessary *High and strong*	Necessary (named lead consultant)	Necessary	Necessary (supervising the team)		✓
**Anesthesiologist**	Necessary (complex obstetrics cases expert) *High and strong*	Recommended (high-resource settings) *High and strong*	Recommended	Necessary	Necessary (obstetrics cases expert)	Recommended	Necessary	Necessary *High and strong*	Necessary	Necessary	Necessary		✓
**Surgeon expert in complex pelvic surgery**	Necessary *High and strong*	Necessary (all settings) *High and strong*	Recommended	Necessary	Necessary	Necessary *Strong*	Necessary *Expert opinion*	Necessary *High and strong*	Necessary	Necessary			✓
**Urologist**	Necessary (open urological surgery expert) *High and strong*		Recommended		Necessary		Should be available on site	Recommended	Recommended	Necessary			✓
**Neonatologist**	Necessary *High and strong*		Recommended	Recommended	Necessary			Necessary *High and strong*	Should be available 24/7	Recommended			✓
**Interventional radiologist**	Necessary *High and strong*		Recommended		Necessary			Recommended	Recommended	Necessary			✓
**Colorectal and vascular surgeons**	Should be available on site						Should be available on site			Necessary *Expert opinion*			⚠
**Hematologist**	Should be available on site				Necessary			Should be available on site *High and strong*	Should be available 24/7	Should be available on site			✓

[a] *Areas with good agreement, considerable disagreement, or insufficient guidance are flagged by a green checkmark, red x, or a black triangle with the exclamation mark, respectively.*

PAS, Placenta Accreta Spectrum; IS-PAS, International Society - Placenta Accreta Spectrum; FIGO, Fédération Internationale de Gynécologie et d'Obstétrique; ACOG, American College of Obstetricians and Gynaecologists; SMFM, Society of Maternal Fetal Medicine; RCOG, Royal College of Obstetricians and Gynaecologists; UK, United Kingdom; SOGC, Society of Obstetricians and Gynaecologists

© 2025 Bonanni G et al. *JAMA Network Open.*

of Canada; AWMF, Association of Scientific Medical Societies in Germany; CNGOF= Collège National des Gynécologues et Obstétriciens Français; ACR, American College of Radiologists; NICE, National Institute for Health and Care Excellence; RANZCOG, Royal Australian and New Zealand College of Obstetricians and Gynaecologists; NWIHP, National Women and Infants Health Programme's; IOG, Institute of Obstetricians and Gynaecologists; MSPE, Ministerio de Salud Publica del Ecuador; FECOLSOG, Federación Colombiana de Obstetricia y Ginecología; CD, Cesarean Delivery;wks, weeks of gestation; MDT, Multi-Disciplinary Team; ICU, Intensive Care Unit; NICU, Neonatal Intensive Care Unit; IR, Interventional Radiologist; OR, Operative Room; MFM, Maternal Fetal Medicine; MIS, Minimally-Invasive Surgery

eTable 8. Summary and comparative analysis of guideline recommendations on cesarean hysterectomy management of PAS disorders. The reported level of evidence is indicated in *italics*.

Topic	IS-PAS (2019)	FIGO (2018)	ACOG + SMFM (USA, 2021)	RCOG (UK, 2018)	SOGC (Canada, 2019)	AWMF (Germany, 2022)	CNGOF (France, 2016)	NWIHP + IOG (Ireland, 2022	MSPE (Ecuador, 2017)	Agreement[a]
**Anaesthesia: regional vs general**			Consider general anesthesia	Anesthetist choice made after consulting woman	Regional may be safer and preferred by patients *Moderate*		Epidural or combined spinal are possible. If major hemorrhagic risk, general is ok to avoid emergency conversion	Neuraxial, general or combined are suitable. Case by case decision is recommended		✗
**Routine pre/intra-operative US to map placental edges**	Recommended where possible *Conflicting*	Recommended in case of conservative treatment (high resource settings) *Moderate*			Appropriate	Appropriate ("if necessary")			Recommended *Expert opinion*	✓
**Skin midline incision**	No evidence of benefit for all. Decision based on placenta location, invasion, risk factors,	If placental superior margin is outside the lower uterine segment (all settings) *Low and weak*	Employed by many. Based on operator judgement. *Insufficient*		Recommended *Weak*			Individualized *Expert opinion*		✗

© 2025 Bonanni G et al. *JAMA Network Open.*

	surgeon's preference *Conflicting*									
**Uterine incision**	Upper segment incision recommended if PAS clearly evident on opening, reasonable if antenatally suspected *Conflicting*		Should avoid the placenta when possible		US-guided *Weak*	Sufficient distance from the placenta / transverse to the fundus		Sufficient distance from the placenta *Expert opinion*	Sufficient distance from the placenta *Expert opinion*	✓
**Tranexamic acid**	Recommended *High and strong*	Recommended[b] (immediately prior to or during CD) *High and strong*	*Insufficient*		Recommended *High and strong*	Must be available		Must be available		✓
**Pre/intra-operative balloon occlusion catheters**	Not recommended for all cases *Insufficient*	Insufficient	*Insufficient*		Insufficient	Acceptable		Acceptable on a case-by-case basis		✗
**Bilateral internal Iliac artery ligation**	Insufficient	Insufficient	Routine use not recommended *Insufficient*		Insufficient					⚠
**Routine ureteric stent placement**	May be beneficial in selected cases *Insufficient*		Case-by-case; include urologist if ureteral involvement suspected *Unclear benefit*	Insufficient	May be beneficial *Insufficient*			Helpful for difficult cases		⚠
**Recommended maternal position**	Lithotomy or leg straight parted *Conflicting*		Dorsal lithotomy		Modified lithotomy *Expert opinion*			Dorsal lithotomy *Expert opinion*		✓
**Placental removal**		Not recommended (all settings) *High and strong*	Not recommended					Not recommended	Not recommended	✓
**Total vs subtotal hysterectomy**	Decision based on invasion, bleeding, stability, expertise, case-by-case *Conflicting*	Total preferred if placenta increta o percreta (all settings) *Low and strong*	Total required in most cases due to lower uterine segment involvement or cervical bleeding							⚠

© 2025 Bonanni G et al. *JAMA Network Open.*

**Planned delayed hysterectomy**	Not recommended *Conflicting*	Considered if extensive pelvic invasion (high resource settings) *Conflicting*	Investigational approach especially if percreta. Pending further data *Insufficient*		Not recommended					⚠
**Deliberate cystotomy and excision of involved bladder**		Acceptable if percreta villous tissue involves the bladder *Conflicting*								⚠

[a] *Areas with good agreement, considerable disagreement, or insufficient guidance are flagged by a green checkmark, red x, or a black triangle with the exclamation mark, respectively.*
[b] *1g slow IV or 1000-1300 mg orally.*

PAS, Placenta Accreta Spectrum; IS-PAS, International Society - Placenta Accreta Spectrum; FIGO, Fédération Internationale de Gynécologie et d'Obstétrique; ACOG, American College of Obstetricians and Gynaecologists; SMFM, Society of Maternal Fetal Medicine; RCOG, Royal College of Obstetricians and Gynaecologists; UK, United Kingdom; SOGC, Society of Obstetricians and Gynaecologists of Canada; AWMF, Association of Scientific Medical Societies in Germany; CNGOF, Collège national des gynécologues et obstétriciens; ACR, American College of Radiologists; NWIHP, National Women and Infants Health Programme's; IOG, Institute of Obstetricians and Gynaecologists; MSPE, Ministerio de Salud Publica del Ecuador; CD, Cesarean Delivery; wks, weeks of gestation; TVU, transvaginal ultrasound

eTable 9. Summary and comparative analysis of guideline recommendations on the conservative management of PAS disorders for women wishing fertility preservation. The reported level of evidence is indicated in *italics*.

Topic	IS-PAS (2019)	FIGO (2018)	ACOG + SMFM (USA, 2021)	RCOG (UK, 2018)	SOGC (Canada, 2019)	AWMF (Germany, 2022)	CNGOF (France, 2016)	NWIHP + IOG (Ireland, 2022)	PSGO (Poland, 2018)	RANZCOG (Australia and New Zeland, 2023)	MSPE (Ecuador, 2017)	FECOLSOG (Colombia, 2023)	Agreement[a]
Counseling for women wishing fertility preservation													
**Intrapartum diagnosis after VD**	Essential diagnostic criteria: no plane of cleavage between myometrium and placenta at the time of manual exploration (at least in focal areas) *Conflicting*		If stable, delay case until optimal surgical expertise arrives		If no easy plane of separation identified via bimanual examination, cut umbilica l cord and ligate short, observe 12-24 hours, transfer patient if needed, discharge home with weekly f-up							Suspicion must be confirmed (e.g., by means of ultrasound) before attempting manual extraction of the placenta	⚠

© 2025 Bonanni G et al. *JAMA Network Open.*

**Intrapartum diagnosis and subsequent management after laparotomy**	Diagnosed confidently if 1) frank signs, 2) “dimple” signs upon gentle placental traction 3) no plane of cleavage upon gentle digital exploration *Conflicting*		Delay case for optimal surgical expertise; alert anesthesia and critical care teams. If uterus already opened, rapid closure and hysterectomy considered.	CD should be delayed until appropriate staff and resources available if clinical stability (or closure and urgent transfer if necessary); if baby delivered, emergency hysterectomy indicated *Expert consensus*	If uterus not incised, close abdomen and transfer to a regional PAS center. Classical CD with closure of the uterus is an option.		Both conservative and hysterectomy approaches are possible *Weak*		Hysterectomy 16ecommen *Conflicting*			CD should be delayed for clinical stability, until appropriate staff and resources are available. If fetal distress, CD and leaving placenta in situ approach until resources are available.	✗
**Counseling for women wishing fertility preservation**													
**Counseling**	Recommended *Moderate*	Recommended	Recommended (explaining all risks)	Recommended (explaining all risks)	Recommended (instruction to access care early in any future pregnancy) *Expert opinion*		Recommended (explaining all risks) *Weak*	Recommended (by senior ob)	Recommended *Weak*	Recommended	Recommended		✓
**Counseling: feasibility of fertility preservation**	May be possible *Moderate*		May be possible *Weak*				May be possible *Weak*		May be possible if patient’s health condition allows *Weak*	It is possible in compliant women under close surveillance	It is possible		✓
**Counseling: risks of massive hemorrhage and possible interventions; risk of urinary tract damage**				Recommended[d]				Recommended		Recommended	Recommended		✓

© 2025 Bonanni G et al. *JAMA Network Open.*

**Counseling: risk of hysterectomy**				Recommended				Recommended	Recommended(chance of preserving uterus is 40-60%)	Recommended	Recommended		✓
**Counseling: risk of ICU admission and death**								Recommended			Recommended		⚠
**Counseling: regional and general anesthesia**				Regional is safe, but transition to general may be needed *Conflicting*				Recommended (as early as possible)			Recommended *Expert opinion*		✓
**Counseling: future pregnancy rate**	86-89%		Uncertain benefits of conservative management *Weak*										⚠
**Counseling: recurrence of PAS**	22-29% *Moderate*	High rates (all resource settings) *High and strong*					High risk						✗
**Counseling: CD scar pregnancy risk**					Increased *Conflicting*								⚠
**Final decision on conservative management**	Only for selected cases (focal disease <50% of anterior wall, no Invasion into parametrium/cervix) *Moderate/weak*		Only for selected cases after detailed counseling *Conflicting*	Only after detailed counseling and if expert surgeon available *Conflicting*	Based on woman's preference (beliefs and needs should be respected)			Should be made at MDT meeting based on woman's preference, disease severity and17ecommended surgical expertise				Only for selected cases and if an expert team is available 24/7	✓
**Surgical approach**													

© 2025 Bonanni G et al. *JAMA Network Open.*

**Extirpative technique / forcible manual placenta removal**	Not recommended *Moderate*	Not recommended (all settings) *High and strong*	Not recommended		Not recommended *Expert consensus*	Not recommended if clear PAS	Not recommended *Conflicting*	Not recommended *Conflicting*		Not recommended			✔
**"Leaving the placenta in situ" approach / expectant management**	Acceptable	Acceptable (in compliant women who agree to long-term monitoring)	Considered only for carefully selected case after detailed counseling *Insufficient*	Acceptable if placenta area is limited and all accessible. Insufficient if percreta.	Acceptable (with close monitoring and quick access to emergency care) – expert note: not offered	Acceptable			Acceptable			Acceptable in selected cases and if an expert team is available 24/7	✔
**One-step conservative surgery (partial myometrial resection)**	If disease is focal, with <50% invasion of the anterior uterine surface.	Less reproducible than other approaches		Feasible if placenta area is limited and all accessible	Possible for focal central disease							If bladder separated, PAS anterior, 2cm healthy myometrium caudal to PAS, >50% uterine circumference intact	✔
**Triple-P procedure**		Insufficient			Possible for focal central disease								⚠
**Gentle attempted removal**	Acceptable if no frank signs of PAS[b] and placenta undisturbed by incision	Possible if suspected false-positive diagnosis (all settings) *Weak*	Uncertain efficacy *Insufficient*	Less acceptable than hysterectomy		If unclear diagnosis					Not recommended. Hysterectomy preferred.		✘
**Methotrexate adjuvant treatment**	Not recommended	Not recommended (high resource settings)	Not recommended *Insufficient*	Not recommended	Not recommended	Not recommended	Not recommended						✔
**Preventive surgical or radiological uterine**	Not recommended (Insufficient)	Not recommended (high		Not recommender (Insufficient)	*Insufficient*	If necessary		Case-by-case MDT decision		Not routinely recommended		Case-by-case MDT decision	✔

© 2025 Bonanni G et al. *JAMA Network Open.*

**devascularization**		resource settings)											
**Aortic balloon vs bilateral iliac occlusion**			*Insufficient*					Aortic balloon preferred					⚠
**Monitoring of leaving the placenta in situ approach**		MRI and/or serum β-hCG not recommended. Insufficient											⚠
						**Hemorrhage management**							
**Life-threating massive hemorrhage during delivery**	Hysterectomy if patient unstable; vascular compression (aorta, common iliacs) to gain time *Conflicting*		1:1:1 to 1:2:4 strategy of packed red blood cells: fresh frozen plasma: platelets *Strong*		Massive transfusion protocol *Expert opinion*	Mechanical autotransfusion if increased blood loss					Massive transfusion protocol *Expert opinion*	In a non-specialist center: hysterectomy, manual aortic compression, telemedicine support	✔
**Manual vascular compression (aorta, common iliac arteries)**	Appropriate in unstable women (to gain time) *Conflicting*		Reserved for heroic measures due to risk of vascular complications									Appropriate if massive bleeding	⚠
**Intrauterine tamponade (e.g., balloon)**	Recommended (first line) if placenta has been removed *Conflicting*				Should be available in OR					May be appropriate if placenta separation occurred *Insufficient*			⚠
**Uterine devascularization (with/without uterine compressive sutures)**	Recommended if intrauterine tamponade fails or if placenta in situ												⚠

© 2025 Bonanni G et al. *JAMA Network Open.*

	Expert opinion											
**Internal iliac artery ligation**	Indicated if persisted PPH after intrauterine tamponade and uterine devascularization in stable women; after hysterectomy in unstable women along with pelvic tamponade[e] *Expert opinion*	May be required *Moderate*	Potential to decrease blood loss, but evidence is inconclusive and method may be ineffective and time-consuming. *Insufficient*		Possible for selected cases *Insufficient*						Possible for selected cases	⚠
**Uterotonics**	Oxytocin is not recommended unless placenta removal or significant bleeding. Administered for significant bleeding per local protocols. *Conflicting evidence*	Not recommended unless no clinical evidence of PAS and attempted placenta removal *(all settings)* *Weak*			Not recommended; should be available in OR *Insufficient*	Recommended *High and strong*						✗
**Hemostatic/procoagulants**	Per local protocols *Conflicting*											⚠
**Tranexamic acid**	Recommended if massive hemorrhage *High and strong*	Recommended (prior or during CD) *High and Strong*	Considered		Recommended (during CD) *High and strong*	Recommended *High and strong*		Considered				✓
**Postoperative antibiotic therapy**	Considered if pelvic tamponade is performed *Conflicting*	Recommended if placenta left in situ[f] (high resource settings) *Weak*	Recommended		Recommended			Necessary				✓

© 2025 Bonanni G et al. *JAMA Network Open.*

[a] *Areas with good agreement, considerable disagreement, or insufficient guidance are flagged by a green checkmark, red x, or a black triangle with the exclamation mark, respectively.*
[b] *Frank signs of PAS after laparotomy: 1) Uterus over the placental bed appears abnormal (can have a bluish/purple appearance) with obvious distension (a "placental bulge"). 2) Placental tissue is seen to have invaded through the surface of the uterus. This may or may not have penetrated the serosa. 3) Excessive, abnormal neo-vascularity is observed in the lower segment (particularly with vessels running craniocaudally in the peritoneum).*
[c] *"Dimple sign": traction on the umbilical cord causes the uterine wall to be visibly pulled inward in the direction of traction without any separation of the placenta.*
[d] *Women diagnosed with placenta accreta spectrum who decline donor blood transfusion should be cared for in a unit with an interventional radiology service.*
[e] *Large steril abnominal swabs and broad spectrum antibiotics while the packing remains in situ.*
[f] *Amoxicillin and clavulanic acid, or clindamycin in case of penicillin allergy.*

PAS, Placenta Accreta Spectrum; IS-PAS, International Society - Placenta Accreta Spectrum; FIGO, Fédération Internationale de Gynécologie et d'Obstétrique; ACOG, American College of Obstetricians and Gynaecologists; SMFM, Society of Maternal Fetal Medicine; RCOG, Royal College of Obstetricians and Gynaecologists; UK, United Kingdom; SOGC, Society of Obstetricians and Gynaecologists of Canada; AWMF, Association of Scientific Medical Societies in Germany; FECOLSOG, Federación Colombiana de Obstetricia y Ginecología; CNGOF= Collège national des gynécologues et obstétriciens; ACR, American College of Radiologists; NWIHP, National Women and Infants Health Programme's; IOG, Institute of Obstetricians and Gynaecologists; PSGO, Polish Society of Gynecologists and Obstetricians; MSPE, Ministerio de Salud Publica del Ecuador; RANZCOG, Royal Australian and New Zealand College of Obstetricians and Gynaecologists; CD, Cesarean Delivery; wks, weeks of gestation; MDT, Multi-Disciplinary Team;TVU, transvaginal ultrasound; PPH: Postpartum hemorrhage.

eTable 10. Summary and comparative analysis of guideline recommendations on postnatal management of PAS disorders. The reported level of evidence is indicated in *italics*.

Topic	SOGC (Canada, 2019)	NWIHP + IOG (Ireland, 2022)	PSGO (Poland, 2018)	MSPE (Ecuador, 2017)	Agreement
**Postnatal analgesia**	Recommended (epidural for 24 hour)	Recommended (management documented)			⚠
**Postnatal referrals**	Recommended to:<br>1) Social work services<br>2) Perinatal mental health	Recommended to:<br>1) Physiotherapy<br>2) Social work services<br>3) Perinatal mental health		Recommended<br>(to first level of care)	✔
**Postnatal visit with specialist care team**	At 6-8 wks	At 6 wks<br>*Expert opinion*			⚠
**Comprehensive patient chart**	Recommended	Recommended (all relevant clinical information and intraoperative findings)		Recommended	✔
**Placental pathology**	Recommended	Recommended (FIGO classification); to be presented at MDT meeting once available	Recommended<br>(final diagnosis)		✔
**Postnatal iron supplementation**	**Note of expert: IV iron usually not needed due to aggressive iron supplementation in the antenatal period*	Recommended for women who were anemic antenatally or with postnatal anemia, for at least 6 weeks postnatally<br>*Moderate evidence*			⚠
**Postnatal thromboembolism prevention**	Recommended (LMWH)	Compression stockings, adequate hydration, early mobilization recommended; thromboprophylaxis based on individual risk			⚠

[a] *Areas with good agreement, considerable disagreement, or insufficient guidance are flagged by a green checkmark, red x, or a black triangle with the exclamation mark, respectively.*

PAS, Placenta Accreta Spectrum; SOGC, Society of Obstetricians and Gynaecologists of Canada; NWIHP, National Women and Infants Health Programme's; IOG, Institute of Obstetricians and Gynaecologists; PSGO, Polish Society of Gynecologists and Obstetricians; MSPE, Ministerio de Salud Publica del Ecuador; CD, Cesarean Delivery; wks, weeks of gestation; MDT, Multi-Disciplinary Team; LMWH, Low Molecular Weight Heparin

© 2025 Bonanni G et al. *JAMA Network Open.*
