# Supplementary material for: Guidelines on Placenta Accreta Spectrum Disorders: A Systematic Review
Source: JAMA Netw Open. 2025 Jul 18;8(7):e2521909. doi: 10.1001/jamanetworkopen.2025.21909 (PMC12274978; doi:10.1001/jamanetworkopen.2025.21909)
Supplement: Supplement 2. — Data Sharing Statement [file jamanetwopen-e2521909-s002.pdf]

Data Sharing Statement

Bonanni. Guidelines on Placenta Accreta Spectrum Disorders. *JAMA Netw Open*. Published July 18, 2025. doi:10.1001/jamanetworkopen.2025.21909

Data
Data available: Yes
Data types: Data (not involving human participants)
How to access data: Supplement 1
When available: With publication

Supporting Documents
Document types: None

Additional Information
Who can access the data: Anyone accessing supplementary files
Types of analyses: N/A
Mechanisms of data availability: N/A
